# Outbreak of carbapenem-resistant Enterobacterales at a long-term care facility in Seoul, Korea: surveillance and intervention mitigation strategies

**DOI:** 10.4178/epih.e2023057

**Published:** 2023-06-09

**Authors:** Yoojin Park, Euncheol Son, Young June Choe, Cho Ryok Kang, Sangmi Roh, Young Ok Hwang, Sung-il Cho, Jihwan Bang

**Affiliations:** 1 Seoul Metropolitan Government, Seoul, Korea; 2 Department of Pharmacology, University of Ulsan College of Medicine, Ulsan, Korea; 3 Department of Pediatrics, Korea University Anam Hospital, Seoul, Korea; 4 College of Nursing, Seoul National University, Seoul, Korea; 5 Eunpyeong Public Health Center, Seoul, Korea; 6 Department of Disease Research, Seoul Institute of Public Health and Environment, Seoul, Korea; 7 Department of Public Health Science, Graduate School of Public Health, Seoul National University, Seoul, Korea; 8 Division of Infectious Diseases, Seoul Metropolitan Government-Seoul National University Boramae Medical Center, Seoul, Korea; 9 Department of Internal Medicine, Seoul National University College of Medicine, Seoul, Korea

**Keywords:** Carbapenem-resistant Enterobacterales, Long-term care, Infection control, Caregivers

## Abstract

**OBJECTIVES:**

Because effective decolonization options are not available, and treatment options are limited, carbapenem-resistant Enterobacterales (CRE) constitute increasingly threatening nosocomial pathogens. To prevent CRE-associated transmission and ensure patient safety, healthcare personnel and everyone in contact with CRE-infected patients must implement stringent infection control practices. This report describes a CRE outbreak, possibly related to a caregiver at a long-term care facility (LTCF), and presents a new surveillance model to improve the infection control of CRE in Seoul, Korea.

**METHODS:**

The Seoul Metropolitan Government surveillance system identified an outbreak of CRE in an LTCF in 2022. We obtained data on the demographic characteristics and contact histories of the inpatients, medical staff, and caregivers. To isolate the inpatients and employees exposed to CRE, we used rectal swab samples and environmental sampling during the study period (May-December 2022).

**RESULTS:**

We identified 18 cluster cases (1 caregiver and 17 inpatients) and 12 sporadic cases with CRE, and conducted a complete 197-day follow-up of all cases in the LTCF’s isolation wards.

**CONCLUSIONS:**

This investigation demonstrated that our surveillance model and targeted intervention, based on the cooperation of the municipal government, public health center, and infection control advisory committee, effectively contained the epidemic at the LTCF. Measures to improve the compliance of all employees in LTCFs with infection control guidelines should also be adopted.

## GRAPHICAL ABSTRACT

[Fig f3-epih-45-e2023057]

## INTRODUCTION

Carbapenem-resistant Enterobacterales (CRE) are increasingly threatening nosocomial pathogens because there are no effective decolonization methods and limited treatment options [1,2]. Notably, all-cause mortality associated with CRE infections among inpatients was estimated to be approximately 40% in the United States and Israel [3].

Over the past decade, several *Klebsiella pneumoniae* carbapenemase (KPC)-producing CRE outbreaks have been reported in acute care facilities in multiple countries, including Korea, the United States, and Greece [4-6]. A previous study in Korea reported that the prevalence of intestinal colonization by carbapenem-resistant organisms and *Enterobacteriaceae* was 17.5% (7/40) and 7.5% (3/40), respectively, in tertiary care hospitals [4]. Moreover, carbapenem resistance is mainly related to KPC, and studies showed that New Delhi metallo-β-lactamase (NDM) was the second dominant carbapenemase from 2011 to 2015 [7,8]. A study of 113 hospitals in Seoul reported that the common CRE types were *Klebsiella pneumoniae* (56.5%) and *Escherichia coli* (17.0%) in 2018. Among the carbapenemase-producing Enterobacterales (CPE) types in the report, 46.0% were KPC-2 and 5.9% were NDM-1 [9].

Long-term care facilities (LTCFs), which provide post-acute care hospitalization for patients with complicated illnesses, play a pivotal role in the spread of CRE within healthcare systems [10]. Between 2007 and 2018 in Korea, the antimicrobial resistance in LTCFs was higher than that in the general population [11]. This might be because most inpatients in LTCFs have multiple underlying diseases and are prescribed a variety of medications (including antibiotics), which makes them vulnerable to colonizing resistant organisms. Another study demonstrated that approximately two-thirds of the patients in LTCFs have histories of antibiotic exposure spanning 65 years or more, making them much more vulnerable to CRE colonization [12]. Thus, infection prevention and the control of CRE outbreaks in LTCFs are of special concern. However, strategies for the surveillance and management of CRE outbreaks in LTCFs remain insufficient [13].

In May 2022, the infectious disease surveillance system operated by the Seoul Metropolitan Government (SMG) warned of a possible CRE outbreak in an LTCF in Seoul. The municipal government, district public health center, and advisory committee on infection control developed a joint response team to conduct epidemiological field investigations. In this report, we describe the results of one epidemiologic field investigation of a CRE outbreak (possibly related to a caregiver), following the Outbreak Reports and Intervention Studies of Nosocomial Infection (ORION) guidelines and a recently conceptualized surveillance model for epidemiological investigation management in Seoul.

## MATERIALS AND METHODS

### Surveillance for carbapenem-resistant Enterobacterales

The infectious disease surveillance system of the SMG monitors outbreaks and operates the systematic collection, analysis, and interpretation of data according to the ORION statement [14]. On May 2, the CRE surveillance team recognized the possibility of a KPC-producing *K. pneumoniae* outbreak at an LTCF. According to the Korea Disease Control and Prevention Agency protocol, all medical facilities, including LTCFs and commercial laboratories in Korea, are obliged to report to the local health authority, such as the SMG, within 24 hours if CRE is detected in clinical samples.

The epidemiological investigation officer affiliated with the local health authority assesses the identified cases and conducts a cluster investigation when 2 or more CPE cases indicate epidemiological relevance based on the time and space of the occurrence as well as the microbiological characteristics. Contact tracing can identify new cases before the onset of clinical symptoms. We defined the reported cases as “sporadic” if they were not epidemiologically related to other cases. The cases in a cluster (“cluster cases”) were classified as “surveillance cases” or “traced cases,” depending on whether they were identified during the surveillance protocol or through contact tracing.

**Figure 1.**
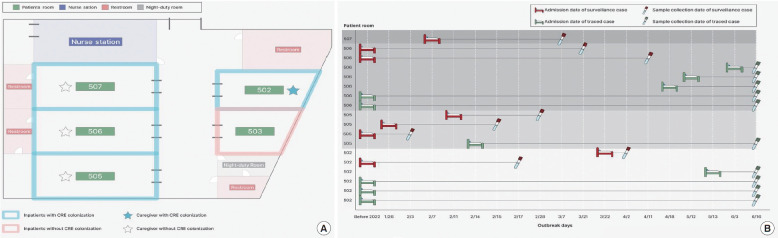
(A) Ward #5 layout in the long-term care facility. (B) Carbapenem-resistant Enterobacterales (CRE) colonization timeline by patient room number. The horizontal line indicates dates of admissions (depicted as beds) and the sampling of specimens for CRE isolation (depicted as specimen tubes). The surveillance system identified 8 cluster cases (depicted as red beds), with an additional 10 cases identified by contact tracing of the cluster cases (depicted as green beds).

The surveillance found that most KPC-producing *K. pneumoniae* colonizers were related to the sporadic cases of CRE in isolation ward #3 and the cluster cases in the intensive care unit (ward #5) at the LTCF. This triggered further on-site investigations to confirm whether these cases were epidemiologically related (Figure 1).

### Epidemiologic investigation

On May 18, the SMG, district public health center, and advisory committee on infection control launched a joint response team to conduct an epidemiological investigation and consult with the LTCF on infection prevention. Our response team investigated any epidemiological associations between the confirmed KPC-producing *K. pneumoniae* cases, including common exposure factors and direct or indirect contact among patients. The history of patient transfers between beds or rooms was identified through the transfer records written by nurses, administration office documents, and interviews with nursing staff. To evaluate the possibility that KPC-producing *K. pneumoniae* was spread via the LTCF staff, we conducted face-to-face interviews with nurses and caregivers, in addition to checking duty schedules and surveillance cultures. A further evaluation was not conducted among the attending physicians because they had had little contact with the patients.

If a CRE was isolated, an additional multiplex polymerase chain reaction for the detection of common carbapenemases (oxacillinase, Guiana extended-spectrum beta lactamase, NDM, Verona integron-encoded metallo-beta-lactamase, KPC, and imipenemase) was performed using the E-9500 CRE Screening Kit (GeNetBio, Daejeon, Korea), according to the manufacturer’s instructions. Contact tracing of CRE-positive patients was conducted at the LTCF, investigating all hospitalized patients, nurses, and caregivers who had histories of both direct and indirect contact with the CRE cases.

We identified all contacts of the ward #5 cluster and offered testing to patients and employees who had been living or working in the same ward as the patient who tested positive for CRE, from January 1, 2022 to May 18, 2022. Confirmed cases of CRE colonization or infection were isolated or grouped together [15].

### Laboratory evaluation

To confirm CRE colonization, rectal swabs were performed for 25 patients who were hospitalized (excluding patients who were discharged during the study period), 6 nurses (excluding those on leave), and 4 caregivers as part of contact tracing in ward #5 on May 18, 2022 and June 15, 2022. Doctors with relatively short stays in the ward were excluded from the study. Follow-up surveillance cultures were performed every 3 weeks to confirm the negative conversion of CRE in this outbreak.

Environmental sampling was also coordinated and performed by the joint response team during the epidemiological field investigation on May 18. Environmental samples, obtained by swabbing surfaces, were collected from the common space, patient rooms, and the nursing station of ward #5. The sites were classified as common spaces (medical waste container, laundry door handle, toilet, washstand, and handle in the shared shower room), patient room #502 (caregiver’s closet and refrigerator handle), patient room #506 (caregiver’s closet, refrigerator handle, and microwave oven handle), patient room #507 (toilet handle), and nurse stations (keyboard, mouse, and stethoscopes). Antimicrobial susceptibility was defined by the interpretative criteria of the Korea Disease Control and Prevention Agency, which was established according to the M100-30th standard of the Clinical and Laboratory Standards Institute and EUCAST guidelines version 12.0 (the European Committee on Antimicrobial Susceptibility Testing, Växjö, Sweden) [16]. If CRE was isolated, a genotypic carbapenemase test was performed as described above.

### Intervention for infection control

**Figure 2.**
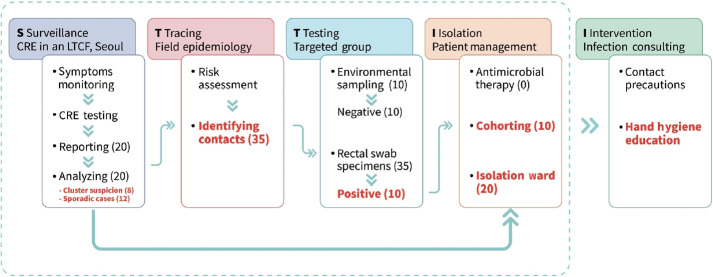
The surveillance logic model applied to a targeted carbapenem-resistant Enterobacterales (CRE) outbreak investigation in Seoul, Korea, 2022. LTCF, long-term care facilities.

Our joint response team conducted an infection prevention consultation after the on-site epidemiological investigation. The consultation team met with the LTCF managers to review their infection control policies and provide infection control training for healthcare personnel, including caregivers. These steps allowed them to contain the spread of CRE. Based on the available data, we wrote an outbreak report according to the ORION protocol, considering the variety of interventions, settings, and logic models pertinent to the LTCF-associated infections (Figure 2).

### Ethics statement

This study was conducted by the SMG in accordance with the mandatory regulations of the Act on Infectious Disease Prevention and Control. Therefore, this study did not require approval from the ethics committee.

## RESULTS

At the time of the outbreak, the LTCF was operating 7 wards with 200 beds, where ward #3 was a designated isolation unit and ward #5 was an intensive care unit. In this intensive care unit, the rooms were separated by a temporary wall that was lower than the height of a person, and the entrance to each room consisted of an open space without a door.

Generally, employees worked in their assigned wards only, and caregivers cared for 3-5 patients in each room. The caregivers worked in the patients’ rooms without any physical or spatial barriers between them and the patients’ beds. Hand sanitizers were placed at the patients’ bedsides and on nursing carts but not at the entrances to the patient rooms. The nurses reported that they practiced hand hygiene every time they entered patients’ rooms.

We identified 30 confirmed CRE cases, of whom 12 (40.0%) were sporadic cases from newly admitted patients, 8 (26.7%) were detected during surveillance, and 10 (33.3%) were traced cases from the ward #5 cluster. Among the 30 patients for whom demographic information was available, 17 (56.7%) were males, and the median age was 76.0 years (interquartile range, 69-83). Most (n=29, 96.7%) of the confirmed cases were inpatients, and 1 (3.3%) was a caregiver. The mortality rate was 16.7% (5/30) and all environmental samples tested negative (Table 1).

Attack rates were calculated for the contacts identified by tracing the confirmed cases. There were 35 contacts (25 inpatients, 4 caregivers, and 6 nurses), among whom 10 were confirmed positive for CRE (9 inpatients and 1 caregiver), resulting in an overall attack rate of 28.6%. The specific attack rates for patient rooms #502, #503, #505, #506, and #507 were 100% (7/7), 0.0% (0/4), 33.3% (3/9), 87.5% (7/8), and 11.1% (1/9), respectively. The patients and caregivers for rooms #502, #505, #506, and #507 were included in the denominator when calculating the attack rate for each room.

The mean time to negative conversion of CRE was 73 days (range, 29-197) in the CRE cases. The caregiver who was positive for CRE worked in patient room #502, and his CRE test result became negative 118 days after the initial positive result. He had no underlying diseases or recent exposure to antibiotics. The other 3 caregivers in rooms #505, #506, and #507 had negative CRE test results. Although he had no other risk factors, the positive caregiver had never received formal infection control training.

Among the 30 CRE-positive patients, 33 CRE bacterial types were detected: 28 (84.8%) *K. pneumoniae*, 2 (6.1%) *Escherichia coli*, 2 (6.1%) *Citrobacter koseri*, and 1 (3.0%) *C. braakii* (Table 1). The most detected carbapenemase genotype was KPC, which accounted for 96.1% (n= 25), followed by NDM (n= 1, 3.9%). In particular, the caregiver was confirmed to have *C. braakii* and *K. pneumoniae*, carbapenemase genotypes KPC-2 and NDM-1, respectively.

## DISCUSSION

This report describes the epidemiological and laboratory investigation of a CRE outbreak at an LTCF in Seoul, Korea, in 2022. We identified 30 patients with confirmed CRE in the LTCF; however, we restricted our results to ward #5, which had confirmed epidemiological relevance with an attack rate of 41.9% (18/43). One notable finding of our investigation was the caregiver infection, which showed that CRE can be extremely contagious in vulnerable group settings such as LTCFs. The trajectory and magnitude of the outbreak suggest that close contact between caregivers and patients may be a high-risk factor for potential CRE transmission. To the best of our knowledge, this is the first study addressing this issue in Korea.

In general, CRE colonizes patients who have severe underlying diseases and prolonged exposure to antibiotics. Because it rarely occurs, we can say that when CRE is isolated in a healthy person, he or she has likely been exposed to CRE repeatedly. Considering the fecal–oral transmission of CRE colonization, repeated CRE exposure reflects poor hand hygiene. The caregivers in this LTCF resided and worked in the same rooms as the patients and were in close contact with the patients for longer periods of time than the medical staff. However, in Korea, most caregivers in LTCFs are foreign workers or day workers hired by family members of the patient and are not familiar with infection control measures. The number of senior citizens in Korea has increased at an unprecedented rate, and the number of patients using LTCFs has inevitably increased, making them more vulnerable to CRE transmission [17,18]. Therefore, it is crucial that the caregivers of inpatients use standard infection control precautions.

Furthermore, LTCFs provide post-acute care hospitalization for patients with complicated illnesses, and they play a pivotal role in the spread of CRE within healthcare systems [10]. Most vulnerable patients in LTCFs have an increased risk of acquiring infections and colonizing CRE because of their multiple underlying diseases and medications [11]. Therefore, surveillance within LTCFs and across all healthcare systems is warranted to identify the risk factors for CRE infection and to measure the impact of the CRE epidemic trajectory.

Based on our new surveillance model, we determined that it was difficult to distinguish between the group of 2 or more cases with confirmed epidemiological relevance (cluster) and the mixture of sporadic cases at the facility. Thus, recognition of an outbreak can be delayed, as it was in this CRE outbreak. For this reason, monitoring and surveillance strategies need to be improved so that it possible to control an outbreak before it becomes widespread.

Another measure critical to the successful containment of the spread of CRE at this LTCF was the infection consultation conducted after the on-site investigation. A previous study showed that communication with the facility should be geared toward refining the existing infection mitigation strategies in response to the transmission risk factors found during the on-site investigation. Our team provided programs and videos on proper hand hygiene and isolation precautions to educate healthcare personnel and caregivers during the outbreak. This study was conceptualized as a surveillance logic model to enable investigators to assess the investigation process [14].

This study had several limitations. First, because this study was conducted at a single LTCF in Seoul, it is difficult to interpret and generalize the results to the entire region of Korea. Second, we could not define the exact time points of the CRE infection in the caregiver. Additionally, a molecular test should be performed to determine whether the CREs of the patients and the caregiver originated from a common ancestral strain. Thus, it was impossible to confirm whether the caregiver was responsible for the transmission of the CRE. However, considering that the patients were confined in beds, the CRE may have been transmitted via indirect contact (i.e., by the hands of medical staff and/or caregivers).

In conclusion, an effective CRE mitigation strategy in a LTCF can be achieved using a CRE surveillance logic model that recognizes the relevance of CRE epidemics and through a targeted intervention based on cooperation between the local government, public health center, and infection control advisory committee. Measures to improve compliance with infection control guidelines by all employees in LTCFs should also be adopted.

## Figures and Tables

**Figure f3-epih-45-e2023057:**
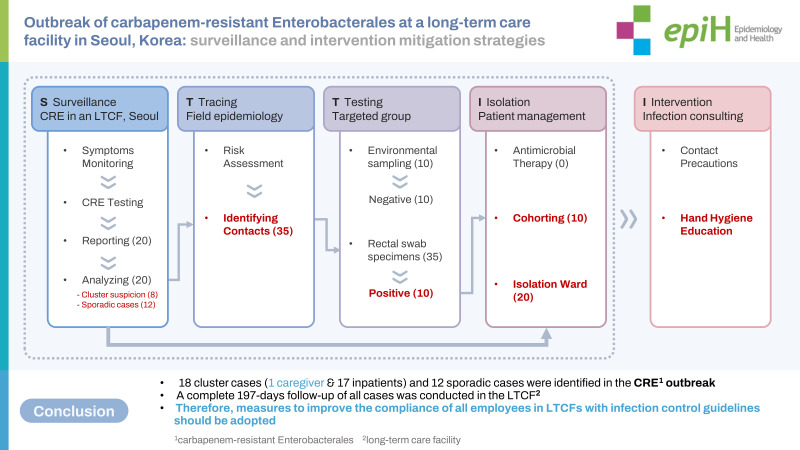


**Table 1.** The general characteristics of CRE colonization among cluster and sporadic cases in Seoul, Korea, 2022

Characteristics	Total	Cluster cases		Sporadic cases
		Surveillance	Traced	
Total	30 (100)	8 (100)	10 (100)	12 (100)
Sex				
Male	17 (56.7)	6 (75.0)	7 (70.0)	4 (33.3)
Female	13 (43.3)	2 (25.0)	3 (30.0)	8 (66.7)
Age (yr)				
Median (IQR)	76.0 (69-83)	78.5 (72-82)	66.0 (57-77)	81.0 (71-85)
50-59	5 (16.7)	1 (12.5)	3 (30.0)	1 (8.4)
60-69	5 (16.7)	-	3 (30.0)	2 (16.6)
70-79	7 (23.3)	3 (37.5)	2 (20.0)	2 (16.6)
80-89	12 (40.0)	4 (50.0)	2 (20.0)	6 (50.0)
90-99	1 (3.3)	-	-	1 (8.4)
Status				
Caregiver	1 (3.3)	-	1 (10.0)	-
Patient	29 (96.7)	8 (100)	9 (90.0)	12 (100)
Room no. (no. of beds+caregiver)				
301 (6+1)	3 (10.0)	-	-	3 (25.1)
305 (6+1)	2 (6.7)	-	-	2 (16.6)
306 (4+1)	2 (6.7)	-	-	2 (16.6)
307 (6+1)	2 (6.7)	-	-	2 (16.6)
308 (6+1)	3 (10.0)	-	-	3 (25.1)
502 (6+1)	7 (23.3)	3 (37.5)	4 (40.0)	-
505 (8+1)	3 (10.0)	2 (25.0)	1 (10.0)	-
506 (7+1)	7 (23.3)	2 (25.0)	5 (50.0)	-
507 (8+1)	1 (3.3)	1 (12.5)	-	-
Pathogens				
*Klebsiella pneumoniae*	28 (84.8)	8 (88.9)	9 (81.8)	11 (84.6)
KPC	23 (82.1)	5 (62.5)	9 (100)	9 (81.8)
Not detected	1 (3.6)	-	-	1 (9.1)
Not performed	4 (14.3)	3 (37.5)	-	1 (9.1)
*Escherichia coli*	2 (6.1)	-	1 (9.1)	1 (7.7)
KPC	1 (50.0)	-	-	1 (100)
Not detected	1 (50.0)	-	1 (100)	-
*Citrobacter koseri*	2 (6.1)	1 (11.1)	-	1 (7.7)
KPC	1 (50.0)	-	-	1 (100)
Not performed	1 (50.0)	1 (100)	-	-
*Citrobacter braakii*	1 (3.0)	-	1 (9.1)	-
NDM	1 (100)	-	1 (100)	-
Death (mortality rate)	5 (16.7)	3 (37.5)	1 (10.0)	1 (8.3)

Values are presented as number (%). CRE, carbapenem-resistant Enterobacterales; KPC, Klebsiella pneumoniae carbapenemase; IQR, interquartile range; NDM, New Delhi metallo-β-lactamase.
